# Dynamics of combatting market-driven epidemics: Insights from U.S. reduction of cigarette, sugar, and prescription opioid consumption

**DOI:** 10.1371/journal.pgph.0003479

**Published:** 2024-07-24

**Authors:** Eszter Rimányi, Jonathan D. Quick, Gavin Yamey, Mustapha Immurana, Vasanti S. Malik, Tanya Doherty, Zain Jafar

**Affiliations:** 1 University of North Carolina at Chapel Hill, Chapel Hill, North Carolina, United States of America; 2 Duke Global Health Institute, Duke University School of Medicine, Durham, North Carolina, United States of America; 3 Institute of Health Research, University of Health and Allied Sciences, Ho, Ghana; 4 Department of Nutritional Sciences, University of Toronto, Toronto, Canada; 5 Health Systems Research Unit, South African Medical Research Council, Cape Town, South Africa; 6 Trinity College, Duke University, Durham, North Carolina, United States of America; University of North Carolina at Chapel Hill, UNITED STATES OF AMERICA

## Abstract

Misuse and overconsumption of certain consumer products have become major global risk factors for premature deaths, with their total costs in trillions of dollars. Progress in reducing such deaths has been slow and difficult. To address this challenge, this review introduces the definition of market-driven epidemics (MDEs), which arise when companies aggressively market products with proven harms, deny these harms, and resist mitigation efforts. MDEs are a specific within the broader landscape of commercial determinants of health. We selected three illustrative MDE products reflecting different consumer experiences: cigarettes (nicotine delivery product), sugar (food product), and prescription opioids (medical product). Each met the MDE case definition with proven adverse health impacts, well-documented histories, longitudinal product consumption and health impact data, and sustained reduction in product consumption. Based on these epidemics, we describe five MDE phases: market expansion, evidence of harm, corporate resistance, mitigation, and market adaptation. From the peak of consumption to the most recent data, U.S. cigarette sales fell by 82%, sugar consumption by 15%, and prescription opioid prescriptions by 62%. For each, the consumption tipping point occurred when compelling evidence of harm, professional alarm, and an authoritative public health voice and/or public mobilization overcame corporate marketing and resistance efforts. The gap between suspicion of harm and the consumption tipping point ranged from one to five decades–much of which was attributable to the time required to generate sufficient evidence of harm. Market adaptation to the reduced consumption of target products had both negative and positive impacts. To our knowledge, this is the first comparative analysis of three successful efforts to change the product consumption patterns and the associated adverse health impacts of these products. The MDE epidemiological approach of shortening the latent time to effective mitigation provides a new method to reduce the impacts of harmful products.

## Introduction

Over the last 50 years, economic development has lifted millions of people from poverty [1], increased child survival and life expectancy [2], and improved human health and wellbeing. Concurrently, the market economy has commercialized a wide range of products whose overuse and misuse have had severe adverse effects on human health and wellbeing [3]. Examples include cigarettes [4–6], sugar [7–11], prescription opioids [12–15], alcohol [16–18], ultra-processed foods [19–21], commercial milk formula (also called infant formula or breast milk substitute) [22–24], firearms [25–27], and social media [28–30]. Each year, such products are significant contributors to the health conditions responsible for more than 850,000 U.S. deaths and 23 million worldwide deaths [31]. The estimated annual global economic losses associated with these products are in the trillions of U.S. dollars [32–35].

Reduction in the human and financial costs of these “market-driven epidemics” (MDEs) has proven highly challenging and typically takes decades to achieve, even when successful. We propose a case definition for MDEs that helps to identify emerging MDEs and address critical barriers to timely, effective prevention and mitigation (Box 1). Key elements of this definition include proof of harm, corporate denial and resistance in the face of established evidence of harm, and effective mitigation efforts. We describe five epidemic phases: market development, evidence of harm, corporate resistance, mitigation, and market adaptation. We apply the MDE definition and show these five phases for three products that reflect different consumer experiences: cigarettes, sugar, and prescription opioids.

**Box 1. Definition of a market-driven epidemic (MDE)**

A market-driven epidemic is a significant increase in the consumption of a consumer product whose overuse or misuse has been proven to cause death, disability, and other harmful effects; whose consumption has been accelerated by aggressive marketing; whose harmful effects are hidden, denied, or otherwise minimized by producers; and for which effective mitigation is actively resisted by producers.

Previous studies of efforts to reduce consumption of potentially harmful products typically have focused on the short-term impact of specific policy interventions [36, 37]. The MDE approach addresses the core epidemiological question: What combination of actions is effective to achieve a sustained, large-scale reduction in the consumption and consequent harms of an MDE product? To the best of our knowledge, this is the first comparative analysis of successful efforts to bend the epidemic curves of consumption and associated adverse health impacts for three distinctive MDEs within one national context (the U.S.).

MDEs represent a specific entity within the broader landscape of the commercial determinants of health (CDoH), defined by the 2023 *Lancet* series as “the systems, practices, and pathways through which commercial actors drive health and equity” [38]. The *Lancet* series mapped commercial sector practices that negatively impact health [38], described commercial entities that go beyond unhealthy consumer products [38], and provided future directions for work in this field [39]. Other CDoH-related work focuses on monitoring corporate political activity [40] and measuring CDoH [41]. The MDE concept adds novel terminology and methodology for bending the epidemic curves of consumption of consumer products and adverse health impacts.

## Methods

### Selection of criteria for illustrative epidemics

We identified three categories of products for this analysis, each meant to represent a distinctly different consumer experience: a nicotine delivery system (cigarettes), a food product (sugar), and a prescription medicine (opioids). Within each category, the specific product was chosen based on the following inclusion criteria: (1) met the case definition for a market-driven epidemic (2) with proven adverse health impacts; (3) well-documented histories of product development, marketing, and mitigation efforts; (4) availability of longitudinal data for product consumption and associated harm; and (5) mitigation efforts that resulted in a significant, sustained reduction in product consumption. Based on these criteria, cigarettes, sugar, and prescription opioids were selected. We used U.S. examples because the U.S. was arguably the epicenter and provided the most robust case histories for each.

### Epidemic curves

The consumption measures for the three epidemic curves respectively were cigarette sales [42], annual consumption of caloric sweeteners (referred to here as “sugar”) [43], and prescription opioids dispensed [44]. The respective associated illustrative health impact measures were adult lung cancer death rate [45], diabetes prevalence rate [46], and prescription opioid overdose mortality rate [47].

### Epidemic phases

The concept of epidemic phases or stages has been used to describe, anticipate, and respond to both infectious and non-infectious outbreaks. Examples include the WHO “continuum of pandemic phases” (interpandemic, alert, pandemic, and transition [48]) and an analysis of the cigarette epidemic in developed countries [49]. For each of the three MDEs, we constructed a detailed timeline of key academic publications, corporate actions, media coverage, legal and regulatory actions, and civil society responses (S1 File). Based on the timelines, epidemic curves, and milestones for the three epidemics, we propose five conceptually distinct, and at times overlapping, phases through which each of the three illustrative epidemics have progressed (Table 1).

**Table 1:** Phases of a market-driven epidemic.

Phase	Description
**1. Market development**	A highly desirable product is created through innovation, i.e. chemical engineering (cigarettes, refined sugar), sometimes transforming an existing product. Aggressive marketing increases product consumption through various strategies that make it more appealing, socially desirable, or addictive, making the product available to a larger population.
**2. Evidence of harm**	Astute clinicians, public health researchers, whistleblowers, or others suspect harm. Diligent epidemiology and clinical research provide compelling evidence confirming harm.
**3. Corporate resistance**	Deaths, illness, and economic and other impacts accumulate. At the same time, companies deny harm, seek to discredit accusers, commission counter-science, “manufacture doubt” with distracting alternative explanations, and mount legal and public relations challenges to mitigation efforts. These corporate tactics aim to forestall action to reduce product consumption and harm.
**4. Mitigation**	A tipping point for concerted action is reached whereby legal, regulatory, political, social, and other mitigation measures are taken at the local, national and/or global levels. Consumption of the MDE product decreases due to the public health forces outweighing the corporate forces that drive consumption.
**5. Market adaptation**	In response to mitigation efforts to reduce consumption, companies and consumers seek alternatives through global expansion (geographic shifts in marketing and consumption), product modification (development of similar or new products), or product substitution (switching company marketing or consumer purchases to alternatives that provide similar psychological or physiological benefits).

## Phases of market-driven epidemics illustrated by cigarettes, sugar, and prescription opioids

### 1) Market development

Market development encompasses the innovation of the MDE product and the ramping up of its production and commercialization. While the cigarette industry pioneered mass marketing [5], the sugar and prescription opioid industries also exemplify methods to popularize, normalize, and grow the market for MDE products.

While tobacco was cultivated for centuries by indigenous Americans and later an integral part of the three-way transatlantic slave trade between Europe, Africa, and the Americas, widespread U.S. cigarette consumption took off after industrialist James “Buck” Duke commercialized cigarettes in the late 1800s [5]. Duke’s American Tobacco Company automated cigarette rolling and made smoking more convenient. Besides engineering mass production, American Tobacco and other cigarette companies developed mass marketing campaigns, notably capitalizing on the two world wars. Companies targeted women as they entered the workforce [50], while soldiers were given cigarette rations to escape the stress of war [51]. The cigarette industry mastered niche appeals using identity marketing to create highly effective advertising campaigns targeting modern women [50, 52], Black Americans [53, 54], and stereotypically masculine white men [55]. Another distinct strategy to increase sales was to make cigarettes even more addictive by increasing the nicotine content [56].

By marketing convenience as a driving factor in the mid-1950s, food corporations created processed food that eliminated cooking and had an increased shelf life [21]. To outcompete other food sellers, corporations hired flavor houses to replace natural ingredients to create similar sensory experiences at a fraction of the cost [57]. Evolutionary psychology was used to increase sales with new flavors and colorful and constantly changing packaging [57]. To maximize consumption, company scientists found the sugar, salt, and fat content “bliss point” [58] and enhanced the textures of food [59]. Food industries marketed aggressively, beginning in schools [60]. In response to the emphasis placed on reduced fat consumption in the 1977 Dietary Goals for the U.S. and other food guidelines, companies introduced products like Nabisco’s “SnackWell’s,” which were low-fat, high-sugar treats that drove refined sugar consumption even higher [61].

In 1986, the World Health Organization (WHO) called for action to address the global challenge of undertreatment of pain due to physicians’ fear of opioid misuse [14, 58]. The industry-influenced ‘Pain as the 5th Vital Sign’ campaign by the American Pain Society in 1995 further set the stage for Purdue Pharma’s creation of OxyContin [14], which launched a year later. Purdue Pharma falsely claimed that OxyContin was a controlled-released opioid with a misuse rate of less than 1% [13]. Building on these claims, Purdue Pharma deployed aggressive marketing tactics. These tactics included sales representatives earning commissions from the amount of opioids prescribed, targeting healthcare workers through infiltrating medical societies and literature, vacation-like conferences, free dinners, merchandise, educational programs, concert and sports event tickets, coupons, grants, and more [13, 62]. Many medical associations also accepted the claims of low rates of misuse, giving painkillers the false facade of safety among doctors. Perhaps the most significant example was The Joint Commission, which tightened pain treatment standards for hospital accreditation in 2000 [63], further supporting the regular prescription of painkillers. While Purdue Pharma was the trailblazer, many companies followed and promoted the opioid crisis, and have since been sued for their efforts, such as CVS, Walgreens, and Walmart [64].

### 2) Evidence of harm

As the market steadily expands for products that ultimately prove to be unhealthy, astute physicians, public health experts, members of the public, or often company staff themselves may raise questions about the product’s effects. Intrigued by anecdotal evidence or individual observations, suspicion of harm is established when a credible source attests to a pattern and authors an initial scientific query, hypothesizing that a product is causing harm to its consumers. Definitive confirmation of harm comes from large, rigorous cohort studies or randomized controlled trials (when ethically feasible). These studies influence the scientific community to reach a consensus that the MDE product is harmful.

While the harms of cigarettes had long been suspected, physician Isaac Adler’s 1912 book-length monograph was the first significant scientific study linking cigarettes to cancers [65]. Four decades later, in 1954, Doll and Hill compared the lung cancer and mortality status of 21,296 smoking to 3,093 nonsmoking doctors over twenty-nine months and concluded that lung cancer rates and mortality increased as the number of cigarettes smoked increased [66]. That same year, the American Cancer Society, the Public Health Cancer Association, and the medical authorities of six other nations recognized that smoking caused lung cancer [4].

For sugar, the journey from suspicion to compelling evidence was more complex. From the 1950s, researchers and clinicians have debated whether the excess intake of sugars [67] or saturated fatty acids (SFAs) [68] was the primary nutritional factor behind the rise of cardiovascular disease (CVD) and other adverse health conditions [7]. Diabetologist John Yudkin’s 1957 *Lancet* article suggested sucrose played an equal or larger role than fats in causing CVD [67]. By 2006, a randomized controlled trial of 48,835 women aged 50 to 79 years showed that intake of refined carbohydrates and added sugars was positively associated with risk of CVD [69]. Norman Temple, a contemporary nutrition researcher, named this fat-sugar-CVD debate "50 years of confusion" [7]. The current consensus is that sugar, particularly through sugar-sweetened beverage (SSB) consumption, is directly linked with CVD but also with weight gain, obesity, and type 2 diabetes [7].

As early as 1997, physicians in rural areas of the U.S., namely Art Van Zee and Vince Stravino, alerted Purdue Pharma, the producer of OxyContin, about rising overdoses. Journalistic reporting amplified these physicians’ reports [70–73]. In 1998, a survey of drug users and dealers found that MS Contin (Purdue Pharma’s extended-release morphine formulation) was one of the most expensive and sought-after street drugs, which users crushed and injected to create a "buzz" [74]. Purdue Pharma executives read this article and noted its findings privately [75]. In 2001, the House of Representatives held a public hearing of Purdue Pharma executives on whether they could have known about and lessened the risk of OxyContin misuse, which the executives denied [76]–their denial contradicts internal documents now publicly available [77]. Despite the attention from government officials and the public, the CDC did not declare an epidemic due to opioid overprescribing until 2011, when it published compelling evidence of the impact of rising use of prescription opioids and overdose deaths [78].

### 3) Corporate resistance

When product safety concerns arise, forward-looking corporate leaders have found pathways to reduce unintended harms. In the mid-2000s, Kraft Foods VP Michael Mudd led an initiative that removed in-school marketing and established nutrition criteria for products sold in school vending machines [79]. In the 2010s, under CEO Indra Nooyi, the sugary drink company Pepsi substantially expanded the proportion of healthy products it sold, while also nearly doubling revenue [80].

Other companies pretend to address the MDE caused by their products and put measures in place for public relations purposes that do not actually lead to decreased consumption. An example from the tobacco industry could arguably be the creation of “light” cigarettes (we discuss product modification in more detail below, under phase 5, market adaptation.) Pretend measures were taken by Purdue Pharma when responding to the FDA’s warnings in the early 2000s, and interestingly, preemptively before OxyContin launched on the market [81]. While we have grouped these measures into corporate resistance, prescription opioids are an example of how the MDE phases are not strictly chronological.

Too often, corporate leaders reflexively defend their products rather than address the risks. Companies have worked to convince consumers and regulators that their products are harmless, even after their internal research finds otherwise. Well-documented corporate resistance tactics include undermining peer-reviewed science, diverting the discourse from their product’s harm, funding civil society groups to voice industry opinions on regulation, and finding legal loopholes to evade regulation [82]. Such tactics undermine consumer protection, prioritizing commercial gains over “protecting health, the environment, or social cohesion” [38]. One of the earliest and most often-used corporate strategies is “manufactured doubt”–deliberate altering and misrepresenting facts and empirical evidence to promote a corporate (or other) agenda [83, 84].

Private corporate research showed that cigarette smoking was carcinogenic as early as 1953 [4] and addictive by 1963 [85]. Yet publicly, the tobacco industry continued to fervently deny these claims and pursue campaigns to increase smoking [85]. Purdue Pharma executives received credible alerts of OxyContin dependence and misuse as early as 1997, yet when testifying to the House of Representatives in 2001, they claimed they had not received data suggesting misuse [76].

Companies have coopted scientific literature by conducting “independent” research to support their products, which were industry-funded without disclosure. In 1954, the Tobacco Industry Research Commission was established to create “controversy” through biased studies on cigarette safety [85]. Similarly, studies funded by the sugar industry’s Sugar Research Foundation suppressed studies that found sugar was more strongly associated with CVD than fat [86]. In the mid-1960s, Harvard University studies with undisclosed sugar industry funding “singled out fat” as the major risk factor for CVD [87]. Industry-funded reviews are especially prone to bias. A 2016 review found that only 1 in 26 industry-funded studies significantly linked SSBs to obesity and diabetes, while 33 out of 34 independently funded studies found adverse health effects of SSBs [88]. Medical opinion leaders paid by the prescription opioid industry misrepresented published evidence to persuade doctors to prescribe opioids at higher doses and longer durations for minor and chronic pain conditions [71, 89].

Many industries have successfully lobbied and funded political campaigns to evade regulations and taxes on their products [90]. In 1977, the sugar industry successfully lobbied for the first U.S. Dietary Guidelines to link CVD primarily to saturated fats, with only a brief mention of reducing sugar consumption [91]. Industry lobbying continued into the 2000s when it attempted–unsuccessfully this time–to block daily sugar calorie limits [92]. Opioid manufacturers have used their funds and influence to obtain favorable treatment in Congressional hearings and bills [72, 93], and to avoid lawsuits [94] and regulation [95]. Purdue Pharma spent $102 million on lobbying the U.S. Congress between 2014 and 2016 [95].

### 4) Mitigation

**Fig 1:**
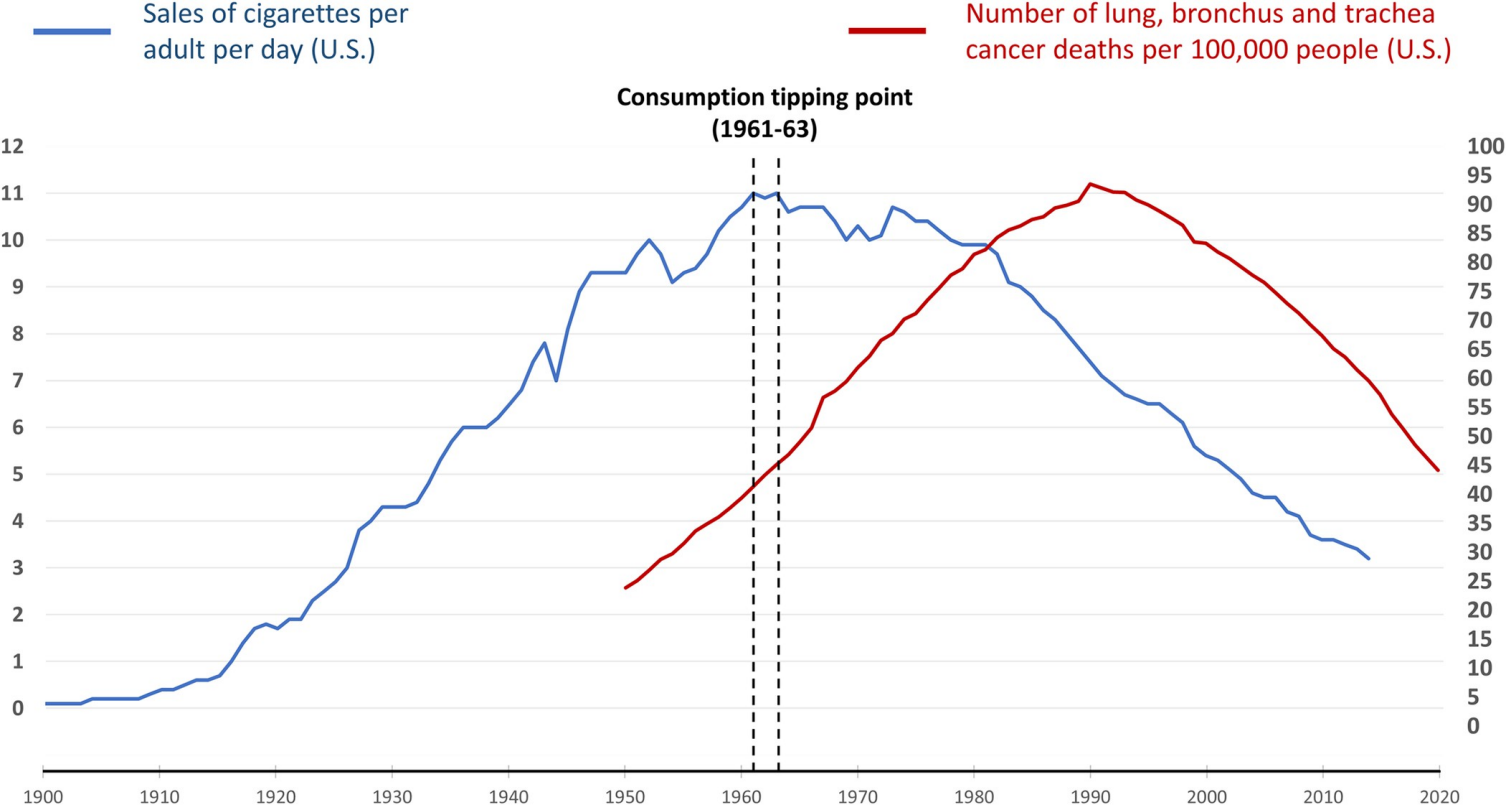
U.S. cigarette sales (1900–2014) vs U.S. lung cancer deaths (1950–2020). Data sources: cigarette sales [42], lung cancer deaths [45].

**Fig 2:**
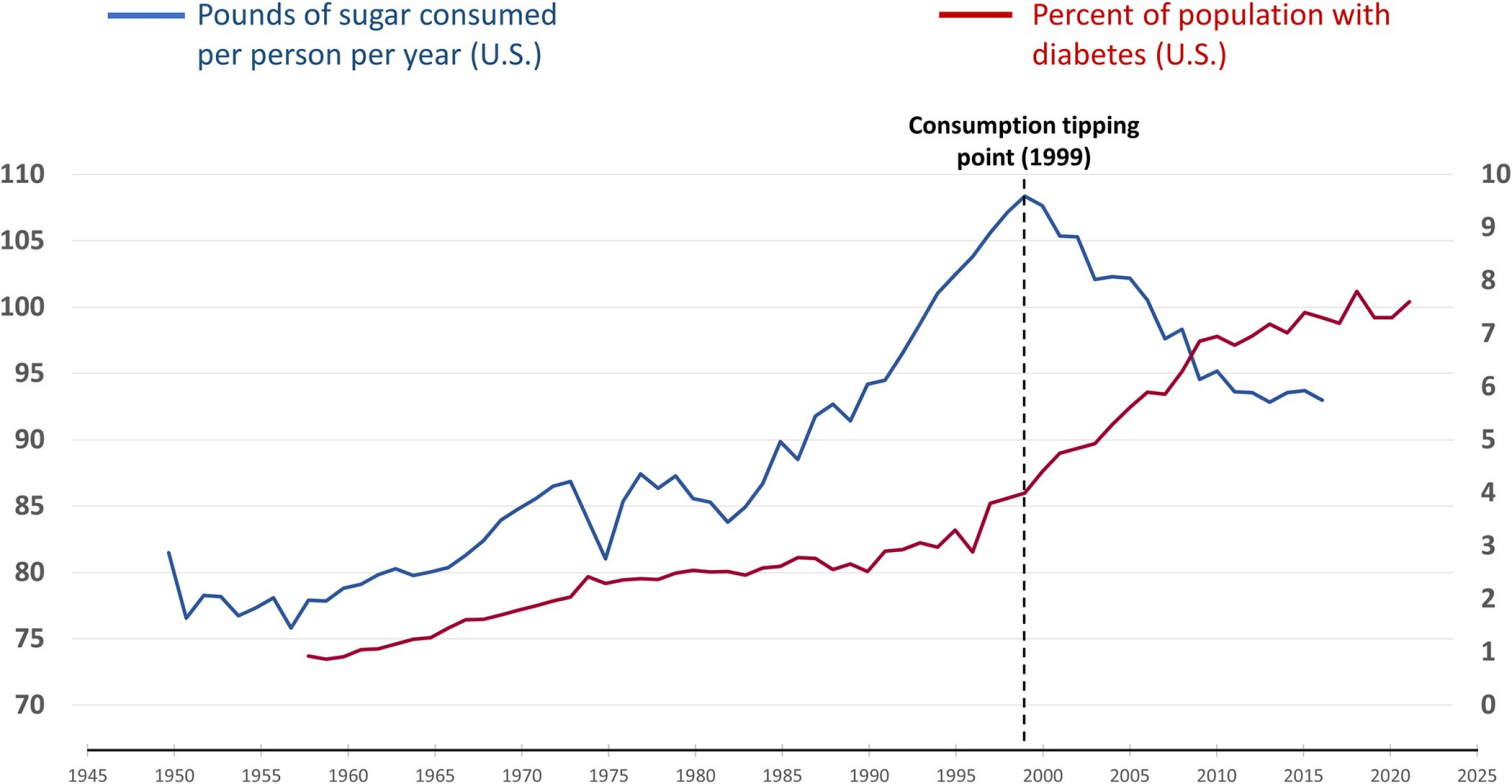
U.S. annual personal sugar consumption (1950–2016). Data sources: sugar consumption [43], diabetes prevalence rate [46].

**Fig 3:**
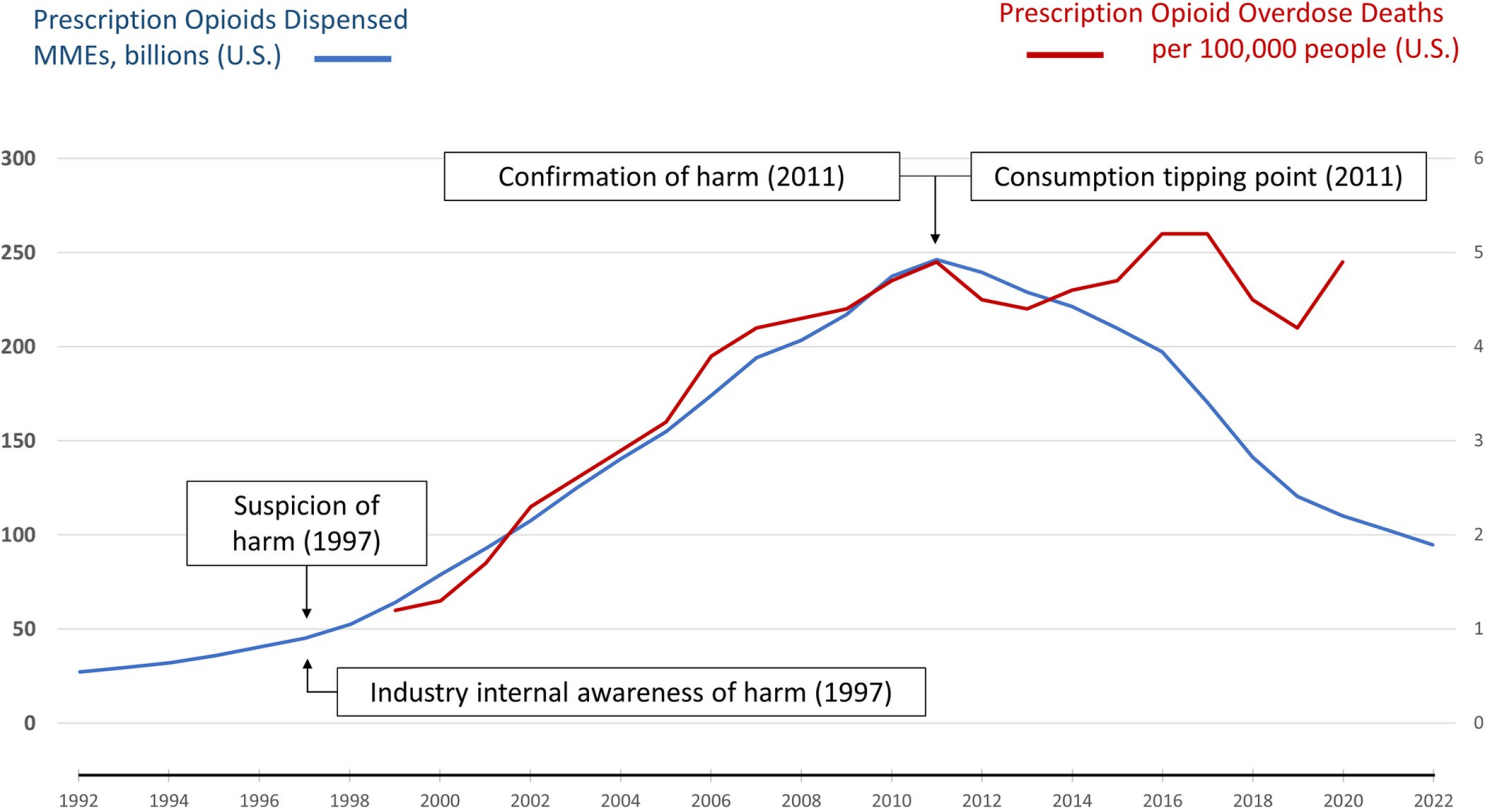
U.S. prescription opioids dispensed (1992–2020) vs U.S. prescription opioid overdose death rate (1999–2022). Data sources: Opioids overdose rates [47], opioid prescription rates [44].

For every epidemic, the core question is: What mitigating actions are required, and by whom, to bend the epidemic curve and thereby reduce or eliminate the resulting illness and death? For infectious diseases, success generally depends on a combination of medical, non-medical, and informational interventions aimed at the infectious agent. For the three subject MDEs, bending the consumption curve in the face of continuing corporate resistance resulted from a combination of actions aimed at changing the behavior of companies and consumers. From the peak of consumption (the “consumption tipping point”) to the most recent data, U.S. cigarette sales fell by 82% from 1963 to 2020 (Fig 1), sugar consumption fell by 15% from 1999 to 2016 (Fig 2), and opioid prescription dispensing fell by 62% from 2011 to 2022 (Fig 3). The magnitude and persistence of these changes in consumer consumption behavior are notable. In each, the consumption tipping point was associated with a catalytic event or series of events.

The U.S. tipping point for cigarette consumption was the period 1961–1963 (Fig 1) when accumulating evidence linking smoking to increasing rates of CVD, cancers, and certain other illnesses led to the first Surgeon General’s Smoking and Health Report. The report hit the country “like a bombshell” as the leading story on newspaper, radio, and television [96]. Despite continued corporate efforts to sow confusion and deny cigarettes’ health harms [83, 97], a series of actions followed the report to drive down consumption, including regulations on package labels and advertisements, federal recognition of nicotine’s addictiveness and secondhand harm, and the creation of public smoking and nonsmoking areas (S1 File). In 1998, the tobacco industry was ultimately held accountable for the harms it knowingly caused when 46 U.S. states won a $206 billion Tobacco Master Settlement agreement against the tobacco industry. By then, cigarette consumption had already fallen to half what it was in 1964 (Fig 1), yet it was still a historic win in getting the industry to reckon with its harms [98].

For sugar, the consumption tipping point was in 1999 (Fig 2). Two key events led to this tipping point. The first was a 1998 landmark publication showing a dramatic increase in obesity in the U.S. from 1960, which raised the alarm about U.S. sugar consumption [99]. The second was a high profile petition launched in 1999 by Science in the Public Interest, called “America: Drowning In Sugar,” endorsed by 72 leading public health organizations and experts, that was aimed at reducing sugar intake [100]. The 2001 Surgeon General’s “Call To Action To Prevent and Decrease Overweight and Obesity” reinforced this aim by encouraging reductions in added sugars [101], and consumption continued to decline (Fig 2) [43]. Randomized controlled trials confirming sugar as a significant CVD risk factor had not yet been conducted, yet individuals quickly and sustainably decreased their sugar consumption (Fig 2).

The consumption tipping point of prescription opioids was in 2011 (Fig 3) when the CDC declared an opioid epidemic due to overprescribing. This declaration was followed by a series of mitigative actions, such as measures ensuring that patients were not being prescribed opioids by more than one doctor [102]. In 2007, Purdue Pharma was federally held accountable for misrepresenting OxyContin’s ability to cause addiction in its users [103, 104]. However, between 2007 and 2011, the amount of OxyContin physicians prescribed continued to increase (Fig 3). Only when the issue of opioid prescription was communicated through a medical channel did physicians realize they had to reduce their prescribing of OxyContin for a wide variety of concerns. In other words, the communication channel must match the audience that decides consumption levels in order to reduce consumption most effectively. Prescription opioid mitigation has continued into recent years, with the litigations brought against the largest opioid manufacturers and pharmaceutical distributors settled and the distributors paying over $25 billion [105].

While one intervention begins the change of consumer behavior, it is often multiple reinforcing actors and actions that shift MDE product consumption over the long-term. Civil society organizations, the media, and internal whistleblowers play catalytic roles in calling out product harm, while government action has been vital in achieving large-scale impacts. Academia plays a central role in confirming or refuting suspected adverse health impacts of consumer products and evaluating the effectiveness of different mitigation strategies. The resulting research publications provide the evidence needed for professional, political, and public support for action. Incontrovertible evidence of harm also helps counter opposition by companies or others skeptical of intervention.

### 5) Market adaptation

While notable progress was made in reducing consumption for the three illustrated products, a substantial level of consumption and associated harm continues. Furthermore, when mitigation efforts began reducing demand for the three MDE products, companies adapted by exploring new markets abroad or through evolving their product. Consumers who reduced their consumption often turned to substitute products, some of which were healthier alternatives and some of which were more harmful.

#### Global expansion

Global expansion occurs when corporations shift product sales, marketing, and consequent harm to less regulated communities and countries in response to a shrinking U.S. market. A 2015 study comparing tobacco marketing in 16 high-, middle-, and low-income countries found that the density of tobacco advertisements was 81 times higher in low-income countries than in high-income countries [106]. Similarly, when U.S. doctors began to reduce their rate of opioid prescribing in 2011, the Sackler-owned Mundipharma implemented Purdue Pharma’s strategies in other parts of the world [107]. For sugar, this pattern is seen particularly in SSB consumption, which is spreading rapidly to low- and middle-income countries, with Latin America and Asia being the prime industry targets [108].

#### Product modification

Companies develop derivative or new products to maintain their sales in the face of decreasing demand for their original product. For example, the tobacco industry has marketed a series of products touted as “healthier,” including filtered cigarettes, low-tar cigarettes, heated tobacco products, and electronic nicotine delivery systems (also called e-cigarettes or vapes, although vapes were developed both within and outside the tobacco industry) [109]. These attempts were marketing schemes for an improved public image, a corporate tactic similar to the manufactured doubt in phase 3.

Responding to the market demand for products with lower sucrose content, food companies turned to high-fructose corn syrup (HFCS), which was perceived as a healthier fruit sugar, and artificial sweeteners. Neither are harmless: HFCS has been associated with CVD and obesity [110, 111], while less consistently, artificial sweeteners have been linked to weight gain and cardiometabolic risk. artificial sweeteners with cancer and CVD [112, 113].

In 2010, Purdue Pharma reformulated OxyContin as its original patent expired, claiming its new version was less prone to misuse. Some studies have concluded that misuse decreased after the reformulation [114], while others suggest that it stayed the same or even increased [115].

#### Product substitution

When consumers become more aware of product harm or when mitigation efforts make products less accessible, they often switch to other products providing similar psychological or physiological benefits. This switch may have positive or negative health consequences. Cigarette-alternative product use continues to grow [116] despite serious questions about its impact on health [117], while substitution of low- and non-caloric alternatives to sugar-sweetened beverages is associated with cardiovascular and other health benefits [118, 119].

In the case of OxyContin, users turned to illicit opiates when they could no longer obtain prescriptions or found that the misuse-deterrent formulation of OxyContin affected their use [114]. While prescription opioid consumption fell after 2011, opioid-related deaths did not. Prescription opioids were the leading cause of opioid overdoses in the period 1990–2010, but heroin-associated overdoses were the leading cause from 2010–2013. Fentanyl then led from 2013 onward [120]. The post-2011 decline in opioid prescriptions may have reduced iatrogenic opioid addiction; however, many who had become addicted to prescription opioids appear to have substituted them with illicit opiates.

## Implications

Where markets and proof of harm are well established, such as the three case examples presented above, public health communities can apply the MDE lens to assess the current state of an MDE, understand local corporate resistance tactics, map current and potential mitigation efforts, and identify potential adverse market adaptations. For emerging global MDE products such as UPFs [121] and social media [28], researchers are still weighing the accumulating evidence of harm [122], concerned corporate leaders are turning to the established corporate playbook [82], and governments are struggling to respond to the unique contexts and challenges presented by each product. Drawing on insight from current MDEs may help navigate the challenges of these and other emerging MDEs.

### Global application of the MDE concept

While the U.S. has achieved significant declines in cigarette, sugar and prescription opioid consumption, the global situation varies for these epidemics. Between 2000 and 2022, the global prevalence of tobacco use among people aged 15 years and older fell by one third, with significant decreases for both men and women and the highest decreases in lower middle-income countries (47%) and low-income countries (43%) [123]. Countries implementing the WHO MPOWER measures developed by the 2003 Framework Convention on Tobacco Control have experienced the largest decreases in tobacco use [124]. Global per capita sugar consumption continues trending upward [116]. SSB consumption, in particular, is rapidly increasing in low- and middle-income countries, led by Latin America and Asia [117]. North America and Western and Central Europe still have the highest opioid consumption rates, while Southeast Asia, Latin America, and Africa have the lowest rates of prescription opioid use [125].

MDEs can originate in any part of the world and emerging MDEs can quickly spread to every part of the world. Violations of the International Code of Marketing of Breast-Milk Substitutes [126]–created in 1981 to counter marketing abuses by commercial milk formula companies–continue today. More children are consuming commercial milk formula than ever before [127] and the estimated annual global child mortality from not breast-feeding is more than 500,000 [128]. Among herbal medicines, which are used by the majority of the world’s population, there are some that may be creating a silent MDE of cancers and other ill effects [129].

### Closing the suspicion-to-evidence gap

In 1997, physicians practicing in rural America alerted the producer of OxyContin to the increase in overdoses related to their product [13], but it was another 14 years before the 2011 CDC epidemiological analysis confirmed their suspicions [78]. More than four decades elapsed between Adler’s 1912 monograph linking cigarettes to cancers [65] and the definitive 1954 Doll and Hill cohort study showing a direct, dose-relationship between smoking and lung cancer mortality [66]. Nearly five decades passed between Yudkin’s 1957 *Lancet* article asserting that sucrose plays a significant role in coronary thrombosis [67] and randomized controlled trials in the mid-2000s definitively confirming the link [69].

Health researchers played a foundational role in generating the evidence of harm and assessment of mitigation strategies that proved invaluable in bending the curves for cigarettes, sugar, and prescription opioids. Longitudinal studies on potentially harmful products take time, and randomized trials for many such products would be unethical [7]. Much of the 42-year suspicion-to-evidence gap for cigarettes is attributable to the lack of rigorous epidemiological tools in the early 1900s. For sugar, however, had there been better research evidence, reducing the 49-year interval between suspicion and confirmation of harm could have averted five decades of misleading dietary advice and potentially millions of premature deaths.

Support from national institutes of health, private foundations, and other non-corporate sources is vital to propel the robust MDE-related research agenda needed to close the suspicion-to-confirmation gap. A process adapted from the WHO R&D Blueprint [130], a tool used for setting investment priorities for pandemic preparedness, could help assess the threat posed by emerging MDEs and set funding priorities.

Reducing the suspicion-to-confirmation interval also depends on ending corporate subversion of the scientific literature and related communications [36]. Existing conflict of interest disclosure requirements [131] and research transparency guidelines [132] are foundational but are not comprehensive [133] and are variably applied by even highly ranked medical journals [132].

Finally, legal requirements for corporate reporting and addressing product harms, as well as consequences for failure to report, might be strengthened where they exist or established in countries and regions where they do not yet exist.

### Reinforcing the role of public health leadership

Trusted, authoritative public health voices effectively communicating the harmful effects of the MDE products mattered greatly in the epidemics presented here. The U.S. Surgeon General’s 1964 smoking report as well as the 2011 CDC declaration of an epidemic of opioid overprescribing created the tipping points for consumption. In both epidemics, public officials were supported by leading academics and professional associations. Anchoring public statements and policies in reliable evidence and communicating the evidence in ways that can be acted on by public health officials and MDE product users is vital to bending consumption curves.

The WHO’s technical assessments, recommendations, and actions continue to play a central role in the global response to MDEs. The WHO’s recommendation that no more than 10% of daily calories should come from free sugars has influenced national recommendations and nutritional labels. The 2003 WHO Framework Convention on Tobacco Control helped provide examples of legal defense against tobacco corporations to countries where tobacco had not yet been a substantial presence. While not all legal advice has been taken, nations have strengthened their laws to defend public health in ways they would likely not have pursued without this code [134].

### Mobilizing mitigation allies

Professional associations and civil society organizations including the Center for Science in the Public Interest, the American Heart Association, National Harm Reduction Coalition, and others played a pivotal role in creating an informed public and changing consumption behavior. On the global level, the NCD Alliance, which works to prevent and control noncommunicable diseases, has a very active role in documenting and combatting “unhealthy commodity industries” [126]. In the U.S., the Truth Initiative arose from the 1998 tobacco Master Settlement Agreement and led the effort that helped reduce the youth smoking rate from 22.6% in 2000 to 2.3% in 2021 [135].

Corporate capture of professional associations has interfered with some associations’ original missions serving public health [136]. For example, the Academy of Nutrition and Dietetics, the world’s largest association of nutrition and dietetics professionals, has been criticized for its financial ties to the food industry, taking “public positions favoring corporations,” and other conflicts of interest [137].

Respected public leaders, journalists, and well-informed pop culture figures play a vital role in communicating proven product harms to the public and amplifying scientific messages with personal stories [70]. Oscar-winning actress Jamie Lee Curtis has commented on her opiate addiction, and Grammy-nominated singer Demi Lovato has made three documentaries about their adverse relationship with alcohol and other drugs [138]. Such personalities are likely to reach youth more effectively than institutional public health messaging.

## Conclusion

Appealing and often addictive products such as cigarettes, sugar, and prescription opioids will continue to be marketed by companies seeking to capitalize on human needs and desires. It is inevitable that some of these products will prove to have serious adverse health impacts. However, the magnitude of illness, death, social disruption, and economic loss caused by overuse and misuse of such products is not inevitable. The magnitude of the noncommunicable disease epidemics demands that public health researchers and practitioners think creatively and apply new lenses to existing issues, as MDEs continue claiming many unnecessary lives. We have written about the successful decreases in MDE product consumption in the U.S., yet public health action is still lagging on in critical areas such as accelerating opioid overdose deaths and rising consumption of HFSS/UPF consumption.

Based on the present analysis, current efforts to combat MDEs will benefit from action in three key areas.

First, the years-to-decades gap between credible suspicion of harm and rigorous confirmation should be closed through research funding priorities based on the threat posed by emerging MDEs, redoubled efforts to prevent corporate undermining of public health research, and exploration of increased reporting requirements for suspected harm.

Second, as in all areas of global public health, the role of public health leadership in combatting MDEs cannot be overstated. Such leadership was a critical element in bending the consumption curves for the three MDEs presented here, as it has been in global efforts to reduce the consumption of harmful products.

Third, mitigation efforts are strengthened and accelerated by mobilizing allies in professional associations, civil society organizations, journalists, and well-informed pop culture figures. Such allies played pivotal roles to bend both the cigarette and the sugar consumption curves. Corporate capture remains a challenge for any potential ally.

While the specific product and circumstances are unique to each MDE, understanding the epidemiology of consumption and health impacts, and epidemic milestones, should help public health leaders combat current MDEs and more swiftly recognize future MDEs. Given the similar patterns among different MDEs, public health leaders, researchers, civil society and others can apply the mitigation strategies presented here to save lives and lessen the impact of continuing and emerging MDEs.

## Supporting information

S1 File Five phase framework events. List of events from tobacco, sugar, and prescription opioids categorized into the five phases of a market-driven epidemic.
(DOCX)
