# Supplementary material for: Dynamics of combatting market-driven epidemics: Insights from U.S. reduction of cigarette, sugar, and prescription opioid consumption
Source: PLOS Glob Public Health. 2024 Jul 24;4(7):e0003479. doi: 10.1371/journal.pgph.0003479 (PMC11268728; doi:10.1371/journal.pgph.0003479)
Supplement: S1 File — List of events from tobacco, sugar, and prescription opioids categorized into the five phases of a market-driven epidemic. (DOCX) [file pgph.0003479.s001.docx]

| **Supplemental File 1. Stages of a Market-Driven Epidemic: Tobacco, Sugar, Opioids** | | | |
| --- | --- | --- | --- |
| **STAGE** | **TOBACCO** | **SUGAR** | **PRESCRIPTION OPIOIDS** |
| 1. **Market development**   *A highly desirable product becomes available to a large population through discovery (e.g., tobacco, sugar) or innovation (e.g., prescription opioids). Aggressive marketing increases product consumption through various strategies that make it more appealing, socially desirable, or addictive.* | **6000 BCE –** Native Americans cultivate tobacco for ceremonial use [1]  **1492 –** Tobacco introduced to Europeans following Christopher Columbus’ voyage to North America [1]  **1531** -- Europeans start cultivation of tobacco in Central America [1]  **1869** - Cigarette manufacturing begins by hand rolling [2]  **1876** – Bonsack develops the first cigarette-rolling machine [3]  **1884** - W. Duke Sons & Company, based in Durham, North Carolina, makes deal with Bonsack for their rolling machine, replacing the need of human rollers [2]  **1886** - W. Duke Sons & Co. sells colorful collectible cards in cigarette packs. [2]  **1890** - W. Duke Sons & Company forces 4 other major cigarette producers to join the American Tobacco Company, controlling 90% of US cigarette market sales [2]  **WWI** **1914-1918** - Cigarettes first donated, then provided, to soldiers on the frontlines. Tobacco companies also market cigarettes to women under the guise of gender liberation and equality.[4] (These same tactics are done under WWII, even more effectively.) Tobacco use increases 346% by 1920. [4]  **1930s** - Niche marketing begins to popularize menthol flavored cigarettes called “Kools” among Black people, sponsors local shops and advertises at culturally Black events and magazines [5]  **1929** - Lucky Strike popularizes cigarettes among women with modern politics and weight-loss promises [4]  **1955** - Marlboro Man campaign launches, targeting men with rugged, outlaw male models [6]  **1987** - Joe Camel cartoon campaign markets specifically to children, to hook a new generation on cigarettes [7] | **Circa 400 BCE** – Sugarcane discovered in India [8]  **1600s-1800s** **–** Sugarcane mass production in Americas [9]  **1850s –** Sugar refined into purified white product with longer shelf life [10]  **1850s-1950s – Appealing, widely available sugary drinks and dessert foods** lead to 8-fold increase in per person sugar consumption [11]  **1950s – General Mills leads marketing shift to convenience** by bringing together processed food companies. [12]  **1980s** - Industrialized food companies start using “variety” as a marketing tool to get consumers to buy more food. This exploits human biology’s idea of satiation. Evolutionarily, people who ate from more variety of food had better nutrition. [13]  **1980s** – Food industry starts producing food with “Bliss point” amount of sugar, that drives increased consumption [13,14] | **1975** – Creation of “McGill Pain Questionnaire,” allowing patients to specify pain experience [15]  **1986** – WHO calls for regular painkiller treatment regimens for cancer and postoperative patients [16]  **1990s** - Professional societies and journals amplify the pain treatment message [17]  **1996** - Purdue introduces OxyContin to the market, stating that their long controlled-release opioid has an abuse rate of less than 1%. [18]  **1996** - Purdue Pharma creates pay system for sale reps where commission is based on milligrams, incentivizing higher doses [19]  **1996-2001** – Purdue hosts 40+ conferences for physicians and nurses on pain management and speaker-training [18]  **Late 1990s- early 2000s** - Physicians begin prescribing OxyContin to nonmajor post-operative patients and patients requesting pain treatment. [18,20].  **1999** - The Veterans Administration hospital system’s introduction of ‘Pain as the 5th Vital Sign’[21]  **2000** - The hospital accreditation by the Joint Commission and funding become dependent on patient satisfactory pain treatment [22]  **2001** - Over 34,000 free coupons redeemed from PP, providing patients with a 7- to 30-day supply of OxyContin  **2002** - Purdue uses marketing data on physicians to identify those with a) highest opioid prescription rate b) those with highest number of chronic pain patients [18,23] |
| 1. **Evidence of harm**   *Astute clinicians, public health researchers, whistleblowers, or others suspect harm. Diligent epidemiology and clinical research provide compelling evidence confirming harm.* | **1912 – Dr. Isaac Adler shows lung cancer cases increase** with increased cigarettes consumption. [24]  **1950 –** First major case control study linking smoking to lung cancer [25]  **1953 --** Smearing smoke tar on mice shown to causes tumors. [26]  **1954** – Landmark Doll and Hill study tying smoking to lung cancer [27]  **1954** - American Cancer Society, the Public Health Cancer Association, and six other nations’ medical authorities adopt the official view that smoking causes lung cancer [3] | **1907** - Physicians hold symposium on increased type 2 diabetes among the rich, possibly due to sugar consumption [28]  **1924** – Epidemiological study links refined sugar intake with diabetes [29]  **1934, 1935** - Elliot Joslin blames diabetes on “overnutrition” and lack of exercise [30,31]  **1954** – Symposium on Coronary Heart Disease causes held as mortality rises [32]  **1957** – John Yudkin’s first major article on sugar [33]  **1960s** - John Yudkin blames added sugars, while Ancel Keys blames fat and cholesterol for CHD [32]  **1972** – Yudkin’s Pure, White and Deadly book is published [34] | **1997 –** Van Zee, Vince Stravino, and other physicians start noticing pattern of OxyContin misuse in users  **1998** – Survey of drug users and sellers in Canada on the street value and use of Purdue Pharma’s MS Contin [35]  **2004** - Article raises concern for patients with chronic pain and history of substance abuse receiving opioid treatment. [36]  **2005** - Report raises concern about opioid oversedation rising [21]  **2005** – Survey of patients in opioid programs shows 89% had misused OxyContin in their lifetime [17] |
| 1. **Corporate resistance**   *Deaths, ill-health, and economic and other impacts accumulate. At the same time, companies deny harm, seek to discredit accusers, commission counter-science, “manufacture doubt” with distracting alternative explanations, and mount legal and public relations challenges to mitigation efforts. These corporate tactics aim to forestall action to reduce product harm.* | **1939** - Philip Morris uses physicians to create science in their favor, advertises in medical journals [37]  **1946** - “More doctors smoke Camels than any other cigarette” slogan poses Camels as the healthiest cigarette [37]  **1954** – Tobacco companies form “The Tobacco Industry Research Commission” to fund studies on tobacco as a public relations strategy. [38]  **1953** - Research done internally at tobacco companies shows that cigarettes are carcinogenic [3]. Executive will testify for decades (into the 1990s) after saying there is no proof for this, only hypotheses. [38]  **1960s** - Companies lower tar to advertise safer cigarettes. Companies employ physicians on ads for “healthier” cigarettes. [39]  **1963** – Research done internally at tobacco companies conclude that nicotine is addictive in addition to carcinogenic [38]  **1972** – ‘Smoking and Health: The Need to Know’ film made by big tobacco is distributed to high school and college students, downplaying carcinogenic effects of smoking [3]  1993 - Philip Morris founds fake grass-roots movement to advocate for smoking in public places [40] | **1943** - The sugar industry creates the Sugar Research Foundation to cast doubt on hazards of sucrose [41]  **1967 –** NEJM review, with undisclosed SRF funding, blames fats and exonerates sugar as CVD risk [41]  **1971** – The Sugar Research Foundation shuts down their studies that indicate that sugar intake raises CHD risk. [41]  **1977 –** The first draft of the U.S. Dietary Goals mentioning sugar is rejected due to pressure from food lobbyists. The revised and republished Dietary Goals for U.S. urges consumers to choose “leaner options” [42]  **1978** - Companies respond to revised dietary guidelines by making “fat-free” alternatives with higher levels of sugar. [42]  **2016** - 2016 review found that only 1 in 26 industry-funded studies significantly linked SSBs to obesity and diabetes, while 33 out of 34 independently funded studies found adverse health effects of SSBs [43] | **Late 1990s-early 2000s**  McKinsey develops a plan for Purdue Pharma to pay “kickbacks” to distributers of OxyContin for every overdose of a prescribed user [44]  **1990s** - The opioid industry funds research by Russell K. Portenoy, consultants, and other researchers called the “Pain Management Movement” to spread notion that opioid treatment of pain does not lead to high levels of misuse [45,46]  **1999** - Purdue Pharma infiltrates medical literature, medical education, and professional medical societies [18]  **2001** – Purdue Executives testify to U.S. Congress that there is not enough evidence on prescription opioid misuse [47]  **2002** – Purdue Pharma funds over 20,000 pain-related educational programs through direct sponsorship or financial grants [48]  **2011** - Lobbyists block legislation requiring specialized training to be able to prescribe painkillers prone to abuse [45] |
| 1. **Mitigation**   *A tipping point for concerted action is reached whereby legal, regulatory, political, social, and other mitigation measures are taken at the local, national and/or global levels. Consumption of the MDE product decreases due to the public health forces outweighing the corporate, consumption-increasing forces.* | **1900-1909** – US State bans on cigarettes enacted, vary from sale of cigarettes to minors to total ban. Enacted due to belief of cigarette smoking being immoral and laziness-inducing [2]  **Mid-1950s-- public perception of cigarettes plummet**, as do the stocks of cigarette manufacturers. [3]  **1964** - The first Surgeon General’s report on smoking formally acknowledges the link between smoking and lung cancer. [49]  **1966** - Congress passes putting health warning on the covers of cigarettes, reading "Caution—cigarette smoking may be hazardous to your health."  **1969** – In response to fairness doctrine, tobacco industry offers to end all television advertising [2]  **1975** – The first statewide law goes into effect that requires separate areas for smoking in public indoor areas (Minnesota Clean Air Act). [50]  **1975** – The military suspends cigarette rations [51]  **1979** - Surgeon general concludes in 12th report on smoking that nicotine is addictive [38]  **1986 –** Health impact to nonsmokers of secondhand smoke officially recognized by U.S. General Surgeon general [50]  **1988** – US Congress prohibits smoking on all domestic flights [50]  **1989** - Surgeon General's report states that cigarette smoking is a major cause of cerebrovascular disease (stroke) [52]  **1992** - EPA recognizes secondhand smoke as a carcinogen, paving the way for more public space bans [53]  **1992** – Nicotine patches are introduced [52]  **1994 –** Leaked Brown & Williamson Tobacco documents revealed the company concealed knowledge of tobacco products causing addiction and cancer. [38]  **1997** - Tobacco industry sued for Joe Camel campaign promoting cigarettes to children [54]  **1998** - California is the first U.S. state to implement a cigarette tax to fund state tobacco control efforts [50]  **1998 -** 46 US states win landmark Master Settlement Agreement against tobacco corporations. The industry is to pay the U.S. States $206 billion in health care damages. TIRC ordered to disband. [39]  **2003 -** WHO Convention on Tobacco Control lists tobacco regulation policy and suggest for all nations [55] | **1984 –** NEJM (where research funded by the sugar industry undisclosed was previously published) now requires disclosure of all conflicts of interest [32]  **1995 -** Sugar Busters! Cut Sugar to Trim Fat book is published [56]. Becomes a bestseller later in 2001.  **1998 -** Flegal et al. paper [57] makes the CDC and medical community aware of the obesity epidemic  **1999 -** Public health response to Flegal paper culminates in a widely endorsed petition to FDA to change sugar labelling, distinguishing between natural and added sugars [58]  **2001 -** Surgeon General's Call to Action to Prevent and Decrease Overweight and Obesity outlines a public health response to reverse widespread overweight and obesity [59]  **2002 –** Multiple NYT cover articles blaming obesity on carbs, also specifically HFCS [60,61]  **2003** – Kraft foods initiative ked by Michael Mudd stops marketing in schools and reduced portion sizes [62]  **2003** - Fat Land bestseller book by Greg Critser criticizes company’s use of cheap HFCS with larger amounts and portions [63]  **2004** – Bray, Nielsen, and Popkin publish the first research article that began the “demonization” of HFCS. [64]  **2005** - Panel formed at UNC from nutritionists to recommend guidelines for SSBs [65]  **2006** – RCT in almost 50k women links refined sugar intake with CHD risk [66]  **2009** - Over 10 million views of a YouTube lecture by Robert Lustig, a pediatrician, on sugar’s effect on the brain [67]  **2010** - Congressional Act reduces sugar in school lunches [68]  **2013** – A study showed bias among research funded by sugar industry, with 83.3% showing inconclusive results on sugar [69]  **2014** - The Healthy Weight Commitment Foundation removes 6.4 trillion calories per year from the U.S. market [70]  **2015 –** U.S. Dietary guidelines advise lowering the consumption of added sugars [71]  **2016 –** Major study finds carbohydrates increase heart disease risk [72]  **2016** - 36% of adults in the United States were obese. [73] | **2001 -** FDA pressures Purdue into adding black box label to OxyContin [45]  **2003 -** FDA states that Purdue Pharma’s ads “omit and minimize the serious safety risks associated with OxyContin” [74]  **2007 –** Purdue Pharma pleads guilty in Federal Court to three felonies, paying $654 million [44]  **2011** – RCT with chronic pain patients finds that escalating dose of prescription has a significant risk of opioid misuse [75]  **2011** – CDC report on opioid overdoses due to over prescribing [76]  **2016** – 49 US states have Prescription Drug Monitoring Programs [77]  **2016** – LA Times articles expose financial incentive system for higher doses of OxyContin and debunks long-acting painkiller theory, prompts Congressional investigation [19]  **2016** - CDC released a “Guideline for Prescribing Opioids for Chronic Pain” for safer opioid use [78]  **2017** – FDA study finds that OxyContin misuse was same or higher after 2010 reformulation plausibly created to avoid patent expiring rather than decrease use [79]  **2017** – US Health and Human Services department announced a 5-point strategy to combat the opioid crisis [80]  **2018 -** Purdue announced that they would not send out sales reps to doctors anymore to promote Oxycontin and was cutting back its sales staff to pre-OxyContin release numbers [81]  **2022** - Over 3000 high level lawsuits against manufacturers, distributors, business advisors, and retailers of prescription opioids [82,83] |
| 1. **Market Adaptation**   *In response to mitigation efforts to reduce consumption, companies and consumers seek alternatives through global expansion (geographic shifts in marketing and consumption), product evolution (development of similar or new products), or product substitution (switching company marketing or consumer buying to alternatives that provide similar psychological or physiological benefits).* | **1960s** - Tobacco’s earliest product evolution was filtered or low-tar cigarettes, which the industry touted as “healthier,” often employing physicians for their advertisements [37].  **1963 - T**obacco companies developed electronic nicotine delivery systems (referred to commonly as e-cigarettes or vapes) [84], which they planned to switch if cigarette smoking declined [84].  **2007 -** Sale of electronic nicotine delivery systems (ENDS) (also called e- cigarettes or vaping products) begins [85]  **2019** – U.S. national data survey found that 1-in-4 high schoolers and a total of 6.2 million middle- and high schoolers were tobacco product users [86], gaining attention from mass media and the U.S. Congress  **2019** - Around 1.1 billion people aged 15 and over smoke, with 80% living in low- and middle-income countries (LMICs) [87]  **2005-2030** - It is estimated that tobacco will have killed 135 million people in the developing world, over 3 times more than in the developed world. [87] | **1984 -** Pepsi and Coca-Cola both start using HFCS instead of sugar [65]  **2014** – Diabetes prevalence is increasing more rapidly in LMICs than in high-income countries (HICs) [88]  **1990-2015** - Sugar-Sweetened Beverage consumption is highest in the Latin America and Caribbean [89]  **1990 – 2017** - Global deaths due to obesity rose from 4.5% to 8% [73]  **2017** – 15% of deaths were attributed to obesity in middle-income countries: high prevalence, but poorer overall health and healthcare systems [73]  **2020** – 80% of CVD deaths occur in LMICs [90] | **2010 –** Purdue Pharma reformulates OxyContin as original patent expires, claiming the new formula is more misuse-resistant [91]  **2013** - Americans turn to deadlier illicit narcotics, such as heroin and fentanyl, after the rate of opioid prescriptions decline in 2012. [92]  **2016** - The Sackler-owned Mundipharma uses the same tactics at Purdue did in the US: awareness campaigns, seminars to doctors, downplaying risks of addiction, video campaigns [93]  **2009 - 2019** – While opioid consumption dropped in the US, Germany, and Canada, it increased in other HICs, upper-middle income countries (UMICs), and LMICs [94] |

# **References:**

1. History of Tobacco in the World — Tobacco Timeline [Internet]. Tobacco-Free Life. [cited 2021 Jun 23]. Available from: https://tobaccofreelife.org/tobacco/tobacco-history/

2. Brandt AM. The Cigarette Century: The Rise, Fall, and Deadly Persistence of the Product That Defined America. Basic Books; 2009.

3. Proctor RN. The history of the discovery of the cigarette-lung cancer link: evidentiary traditions, corporate denial, global toll. Tob Control. 2012;21:87–91.

4. Amos A, Haglund M. From social taboo to “torch of freedom”: the marketing of cigarettes to women. Tob Control. 2000;9:3–8.

5. Wailoo K. Pushing Cool: Big Tobacco, Racial Marketing, and the Untold Story of the Menthol Cigarette. University of Chicago Press; 2021.

6. The Stanford Research into the Impact of Tobacco Advertising (SRITA). Marlboro Men [Internet]. Stanford University. [cited 2024 Jan 18]. Available from: https://tobacco.stanford.edu/cigarettes/filter-safety-myths/marlboro-men/

7. DiFranza JR, Aisquith BF. Does the Joe Camel campaign preferentially reach 18 to 24 year old adults? Tobacco Control. 1995;4:367–71.

8. Johnson RJ, Sánchez-Lozada LG, Andrews P, Lanaspa MA. Perspective: A Historical and Scientific Perspective of Sugar and Its Relation with Obesity and Diabetes1234. Adv Nutr. 2017;8:412–22.

9. Austen RA, Smith WD. Private Tooth Decay as Public Economic Virtue: The Slave-Sugar Triangle, Consumerism, and European Industrialization. Social Science History. 1990;14:95–115.

10. Taubes G. The Case Against Sugar. 2016.

11. Guyenet S. By 2606, the US Diet will be 100 Percent Sugar [Internet]. Whole Health Source. 2012 [cited 2022 Jun 9]. Available from: http://wholehealthsource.blogspot.com/2012/02/by-2606-us-diet-will-be-100-percent.html

12. Hyman DM. Food: What the Heck Should I Eat? Little, Brown; 2018.

13. Moss M. Chapter Five: Variety Seekers. Hooked: Food, Free Will, and How the Food Giants Exploit Our Addictions. Random House Publishing Group; 2021. p. 113.

14. Moskowitz HR, Gofman A. Selling Blue Elephants: How to make great products that people want BEFORE they even know they want them. Pearson Education; 2007.

15. Melzack R. The McGill Pain Questionnaire: Major properties and scoring methods: Pain. 1975;1:277–99.

16. WHO. Cancer Pain Relief. Monograph. 1986;79.

17. Bernard SA, Chelminski PR, Ives TJ, Ranapurwala SI. Management of Pain in the United States—A Brief History and Implications for the Opioid Epidemic. Health Services Insights. 2018;11:1178632918819440.

18. Van Zee A. The Promotion and Marketing of OxyContin: Commercial Triumph, Public Health Tragedy. Am J Public Health. 2009;99:221–7.

19. Ryan H, Girion L, Glover S. ‘You want a Description of Hell?’ OxyContin’s 12-Hour Problem. Los Angeles Times [Internet]. 2016 [cited 2022 Aug 25]; Available from: http://www.latimes.com/projects/la-me-oxycontin-full-coverage/

20. Ahari S. I was a drug rep. I know how pharma companies pushed opioids. Washington Post [Internet]. 2019 Nov 26 [cited 2024 Jan 19]; Available from: https://www.washingtonpost.com/outlook/i-was-a-drug-rep-i-know-how-pharma-companies-pushed-opioids/2019/11/25/82b1da88-beb9-11e9-9b73-fd3c65ef8f9c_story.html

21. Vila HJ, Smith RA, Augustyniak MJ, Nagi PA, Soto RG, Ross TW, et al. The Efficacy and Safety of Pain Management Before and After Implementation of Hospital-Wide Pain Management Standards: Is Patient Safety Compromised by Treatment Based Solely on Numerical Pain Ratings? Anesthesia & Analgesia. 2005;101:474–80.

22. Fenton JJ, Jerant AF, Bertakis KD, Franks P. The cost of satisfaction: a national study of patient satisfaction, health care utilization, expenditures, and mortality. Arch Intern Med. 2012;172:405–11.

23. Stolberg SG, Gerth J. High-tech stealth being used to sway doctor prescriptions. N Y Times Web. 2000;A1, A22.

24. Adler I. Primary Malignant Growths of the Lungs and Bronchi [Internet]. London, UK: Longmans; 1912. Available from: https://www-sciencedirect-com.libproxy.lib.unc.edu/science/article/pii/S0140673657906141

25. Doll R, Hill AB. Smoking and carcinoma of the lung; preliminary report. Br Med J. 1950;2:739–48.

26. Wynder EL, Graham EA, Croninger AB. Experimental Production of Carcinoma with Cigarette Tar. Cancer Res. 1953;13:855–64.

27. Doll R, Hill AB. The Mortality of Doctors in Relation to Their Smoking Habits. Br Med J. 1954;1:1451–5.

28. Charles R, Fernando H, Mallick I, Sandwith P, Ziemann H. Diabetes in the tropics. British Medical Journal; 1907. p. 1059–62.

29. Emerson H, Larimore LD. Diabetes mellitus: a contribution to its epidemiology based chiefly on mortality statistics. Archives of Internal Medicine. 1924;34:585–630.

30. Joslin EP, Dublin LI, Marks HH. Studies in diabetes mellitus. 2. Its incidence and the factors underlying its variations. American Journal of Medical Sciences. 1934;187:433–57.

31. Joslin EP, Dublin LI, Marks HH. Studies in diabetes mellitus. 3. Interpretation of the variations in diabetes incidence. American Journal of Medical Sciences. 1935;189:163–92.

32. Kearns CE, Schmidt LA, Glantz SA. Sugar Industry and Coronary Heart Disease Research: A Historical Analysis of Internal Industry Documents. JAMA Internal Medicine. 2016;176:1680–5.

33. Yudkin J. Diet and Coronary Thrombosis: Hypothesis and Fact. The Lancet. 1957;270:155–62.

34. Yudkin J. Pure, White and Deadly: How Sugar Is Killing Us and What We Can Do to Stop It. London: Davis-Poynter; 1972.

35. Sajan A, Corneil T, Grzybowski S. The street value of prescription drugs. CMAJ. 1998;159:139–42.

36. Passik SD, Kirsh KL. Opioid Therapy in Patients with a History of Substance Abuse. CNS Drugs. 2004;18:13–25.

37. Gardner MN, Brandt AM. “The doctors’ choice is America’s choice”: the physician in US cigarette advertisements, 1930-1953. Am J Public Health. 2006;96:222–32.

38. Glantz SA, Barnes DE, Bero L, Hanauer P, Slade J. Looking through a keyhole at the tobacco industry. The Brown and Williamson documents. JAMA. 1995;274:219–24.

39. Ruegg TA. Historical Perspectives of the Causation of Lung Cancer. Glob Qual Nurs Res. 2015;2:2333393615585972.

40. University of Bath. Astroturfing [Internet]. TobaccoTactics. 2022 [cited 2024 Feb 12]. Available from: https://tobaccotactics.org/article/astroturfing/

41. Kearns CE, Apollonio D, Glantz SA. Sugar industry sponsorship of germ-free rodent studies linking sucrose to hyperlipidemia and cancer: An historical analysis of internal documents. PLoS Biol. 2017;15:e2003460.

42. Oppenheimer GM, Benrubi ID. McGovern’s Senate Select Committee on Nutrition and Human Needs Versus the: Meat Industry on the Diet-Heart Question (1976–1977). Am J Public Health. 2014;104:59–69.

43. Schillinger D, Tran J, Mangurian C, Kearns C. Do Sugar-Sweetened Beverages Cause Obesity and Diabetes? Industry and the Manufacture of Scientific Controversy. Ann Intern Med. 2016;165:895–7.

44. The U.S. Department of Justice. Opioid Manufacturer Purdue Pharma Pleads Guilty to Fraud and Kickback Conspiracies [Internet]. Office of Public Affairs. 2020 [cited 2022 Jul 10]. Available from: https://www.justice.gov/opa/pr/opioid-manufacturer-purdue-pharma-pleads-guilty-fraud-and-kickback-conspiracies

45. Meier B. Pain Killer: An Empire of Deceit and the Origin of America’s Opioid Epidemic. Random House Publishing Group; 2018.

46. McGreal C. Doctor who was paid by Purdue to push opioids to testify against drugmaker. The Guardian [Internet]. 2019 Apr 10 [cited 2024 Jan 22]; Available from: https://www.theguardian.com/us-news/2019/apr/10/purdue-opioids-crisis-doctor-testify-against-drugmaker

47. OxyContin: Its Use and Abuse [Internet]. Pennsylvania, U.S.: United States House of Representatives; 2001. p. 88. Available from: https://www.govinfo.gov/content/pkg/CHRG-107hhrg75754/pdf/CHRG-107hhrg75754.pdf

48. Chakradhar S, Ross C. The history of OxyContin, told through unsealed Purdue documents. STAT [Internet]. 2019 Dec 3 [cited 2023 Jul 27]; Available from: https://www.statnews.com/2019/12/03/oxycontin-history-told-through-purdue-pharma-documents/

49. United States. Surgeon General’s Advisory Committee on Smoking and Health. Smoking and Health: Report of the Advisory Committee to the Surgeon General of the Public Health Service. U.S. Department of Health, Education, and Welfare, Public Health Service; 1964.

50. American Lung Association. Tobacco Control Milestones [Internet]. State of Tobacco Control. 2022 [cited 2022 Sep 12]. Available from: https://www.lung.org/research/sotc/tobacco-timeline

51. Smith EA, Malone RE. “Everywhere the Soldier Will Be”: Wartime Tobacco Promotion in the US Military. Am J Public Health. 2009;99:1595–602.

52. Stratton K, Shetty P, Wallace R, Bondurant S. Time Line of Tobacco Events [Internet]. National Academies Press (US); 2001 [cited 2023 Apr 21]. Available from: https://www.ncbi.nlm.nih.gov/books/NBK222369/

53. Office on Smoking and Health. Control of Secondhand Smoke Exposure [Internet]. Atlanta United States: Centers for Disease Control and Prevention (US); 2006. Available from: https://www.ncbi.nlm.nih.gov/books/NBK44326/

54. Elliott S. Joe Camel, a Giant in Tobacco Marketing, Is Dead at 23. The New York Times [Internet]. 1997 Jul 11 [cited 2024 Feb 12]; Available from: https://www.nytimes.com/1997/07/11/business/joe-camel-a-giant-in-tobacco-marketing-is-dead-at-23.html

55. Zhou SY, Liberman JD, Ricafort E. The impact of the WHO Framework Convention on Tobacco Control in defending legal challenges to tobacco control measures. Tob Control. 2019;28:s113–8.

56. Steward HL, Andrews SS, Bethea MC, Balart LA. Sugar Busters!: Cut Sugar to Trim Fat. Random House Publishing Group; 1995.

57. Flegal KM, Carroll MD, Kuczmarski RJ, Johnson CL. Overweight and obesity in the United States: prevalence and trends, 1960-1994. Int J Obes Relat Metab Disord. 1998;22:39–47.

58. Jacobson MF. Petition for Proposed Rulemaking to Establish a Daily Reference Value for “Added Sugars,” to Require Nutrition Labeling of “Added Sugars,” and to Make Corresponding Changes to Nutrient Content and Health Claim Regulations [Internet]. Washington, D.C.: Center for Science in the Public Interest; 1999 Aug p. 63. Available from: https://www.cspinet.org/sites/default/files/media/documents/resource/sugar-petition-1999.pdf

59. Office of the Surgeon General (US), Office of Disease Prevention and Health Promotion (US), Centers for Disease Control and Prevention (US), National Institutes of Health (US). The Surgeon General’s Call To Action To Prevent and Decrease Overweight and Obesity [Internet]. Rockville, MD: U.S. Department of Health and Human Services; 2001 [cited 2023 Sep 4]. Available from: http://www.ncbi.nlm.nih.gov/books/NBK44206/

60. Pollan M. When a Crop Becomes King. The New York Times [Internet]. 2002 Jul 19 [cited 2023 Mar 22]; Available from: https://www.nytimes.com/2002/07/19/opinion/when-a-crop-becomes-king.html

61. Taubes G. What if It’s All Been a Big Fat Lie? The New York Times [Internet]. 2002 Jul 7 [cited 2023 Aug 4]; Available from: https://www.nytimes.com/2002/07/07/magazine/what-if-it-s-all-been-a-big-fat-lie.html

62. Barboza D. Kraft Plans to Rethink Some Products to Fight Obesity. The New York Times [Internet]. 2003 Jul 2 [cited 2023 Jul 28]; Available from: https://www.nytimes.com/2003/07/02/business/kraft-plans-to-rethink-some-products-to-fight-obesity.html

63. Critser G. Fat Land: How Americans Became the Fattest People in the World. Houghton Mifflin Harcourt; 2004.

64. Bray GA, Nielsen SJ, Popkin BM. Consumption of high-fructose corn syrup in beverages may play a role in the epidemic of obesity. The American Journal of Clinical Nutrition. 2004;79:537–43.

65. Warner M. A Sweetener With a Bad Rap. The New York Times [Internet]. 2006 Jul 2 [cited 2023 Sep 4]; Available from: https://www.nytimes.com/2006/07/02/business/yourmoney/02syrup.html

66. Howard BV, Van Horn L, Hsia J, Manson JE, Stefanick ML, Wassertheil-Smoller S, et al. Low-fat dietary pattern and risk of cardiovascular disease: the Women’s Health Initiative Randomized Controlled Dietary Modification Trial. JAMA. 2006;295:655–66.

67. Sugar: THE BITTER TRUTH [Internet]. 2009 [cited 2023 Aug 4]. Available from: https://www.youtube.com/watch?v=dBnniua6-oM

68. Food and Nutrition Service. Healthy Hunger-Free Kids Act [Internet]. U.S. Department of Agriculture. 2013 [cited 2022 Jun 1]. Available from: https://www.fns.usda.gov/cn/healthy-hunger-free-kids-act

69. Bes-Rastrollo M, Schulze MB, Ruiz-Canela M, Martinez-Gonzalez MA. Financial conflicts of interest and reporting bias regarding the association between sugar-sweetened beverages and weight gain: a systematic review of systematic reviews. PLoS Med. 2013;10:e1001578; dicsussion e1001578.

70. Ng SW, Slining MM, Popkin BM. The Healthy Weight Commitment Foundation Pledge: Calories Sold from U.S. Consumer Packaged Goods, 2007–2012. American Journal of Preventive Medicine. 2014;47:508–19.

71. Dietary Guidelines for Americans. History of the Dietary Guidelines [Internet]. U.S. Department of Agriculture. 2023 [cited 2023 Sep 20]. Available from: https://www.dietaryguidelines.gov/about-dietary-guidelines/history-dietary-guidelines#

72. Grasgruber P, Sebera M, Hrazdira E, Hrebickova S, Cacek J. Food consumption and the actual statistics of cardiovascular diseases: an epidemiological comparison of 42 European countries. Food Nutr Res. 2016;60:31694.

73. Ritchie H, Roser M. Obesity. Our World in Data [Internet]. 2017 [cited 2022 May 18]; Available from: https://ourworldindata.org/obesity

74. Research C for DE and. Timeline of Selected FDA Activities and Significant Events Addressing Opioid Misuse and Abuse. FDA [Internet]. 2022 [cited 2022 Sep 2]; Available from: https://www.fda.gov/drugs/information-drug-class/timeline-selected-fda-activities-and-significant-events-addressing-opioid-misuse-and-abuse

75. Naliboff BD, Wu SM, Schieffer B, Bolus R, Pham Q, Baria A, et al. A randomized trial of 2 prescription strategies for opioid treatment of chronic nonmalignant pain. J Pain. 2011;12:288–96.

76. Centers for Disease Control and Prevention (CDC). Vital signs: overdoses of prescription opioid pain relievers---United States, 1999--2008. MMWR Morb Mortal Wkly Rep. 2011;60:1487–92.

77. Manasco AT, Griggs C, Leeds R, Langlois BK, Breaud AH, Mitchell PM, et al. Characteristics of state prescription drug monitoring programs: a state-by-state survey. Pharmacoepidemiol Drug Saf. 2016;25:847–51.

78. Frieden TR, Houry D. Reducing the Risks of Relief — The CDC Opioid-Prescribing Guideline. New England Journal of Medicine. 2016;374:1501–4.

79. Jones CM, Muhuri PK, Lurie PG. Trends in the Nonmedical Use of OxyContin, United States, 2006 to 2013. Clin J Pain. 2017;33:452–61.

80. Affairs (ASPA) AS of P. What is the U.S. Opioid Epidemic? [Internet]. HHS.gov. 2017 [cited 2021 Sep 20]. Available from: https://www.hhs.gov/opioids/about-the-epidemic/index.html

81. Wight P. Doctors In Maine Say Halt In OxyContin Marketing Comes “20 Years Late.” NPR [Internet]. 2018 Feb 13 [cited 2023 May 24]; Available from: https://www.npr.org/sections/health-shots/2018/02/13/585402385/doctors-in-maine-say-halt-in-oxycontin-marketing-comes-20-years-late

82. Teva agrees to tentative $4.25 billion national settlement over opioids. Washington Post [Internet]. [cited 2022 Jul 27]; Available from: https://www.washingtonpost.com/health/2022/07/27/teva-reaches-opioid-settlement/

83. McKinsey, adviser to businesses around the world, agrees to pay $573.9 million to settle charges for its role in opioid epidemic. Washington Post [Internet]. [cited 2022 Sep 2]; Available from: https://www.washingtonpost.com/business/2021/02/04/mckinsey-opioid-settlement-purdue/

84. Dutra LM, Grana R, Glantz SA. Philip Morris research on precursors to the modern e-cigarette since 1990. Tobacco Control. 2017;26:e97–105.

85. O’Connor R, Schneller LM, Felicione NJ, Talhout R, Goniewicz ML, Ashley DL. Evolution of tobacco products: recent history and future directions. Tobacco Control. 2022;31:175–82.

86. CDC Newsroom. 6.2 Million middle and high school students used tobacco products in 2019: E-cigarettes most commonly used tobacco product [Internet]. CDC. 2019 [cited 2023 May 3]. Available from: https://www.cdc.gov/media/releases/2019/1205-nyts-2019.html

87. Tobacco and the developing world [Internet]. Action on Smoking and Health. 2019 [cited 2022 Aug 4]. Available from: https://ash.org.uk/information-and-resources/fact-sheets/economics-regulation/tobacco-and-the-developing-world/

88. World Health Organization. Diabetes Key Facts [Internet]. 2023 [cited 2024 Feb 13]. Available from: https://www.who.int/news-room/fact-sheets/detail/diabetes

89. Malik VS, Hu FB. The role of sugar-sweetened beverages in the global epidemics of obesity and chronic diseases. Nat Rev Endocrinol. 2022;18:205–18.

90. Prabhakaran D, Anand S, Watkins D, Gaziano T, Wu Y, Mbanya JC, et al. Cardiovascular, respiratory, and related disorders: key messages from Disease Control Priorities, 3rd edition. Lancet. 2018;391:1224–36.

91. Associated Press. Revamped OxyContin was supposed to reduce abuse, but has it? [Internet]. NBC News. 2019 [cited 2024 Jan 24]. Available from: https://www.nbcnews.com/health/health-news/revamped-oxycontin-was-supposed-reduce-abuse-has-it-n1032346

92. CDC. Understanding the Opioid Overdose Epidemic [Internet]. 2021 [cited 2021 Sep 22]. Available from: https://www.cdc.gov/opioids/basics/epidemic.html

93. Ryan H, Girion L, Glover S. OxyContin goes global — “We’re only just getting started.” LA Times [Internet]. 2016 Dec 18 [cited 2022 Aug 4]; Available from: http://www.latimes.com/projects/la-me-oxycontin-part3/

94. Jayawardana S, Forman R, Johnston-Webber C, Campbell A, Berterame S, Joncheere C de, et al. Global consumption of prescription opioid analgesics between 2009-2019: a country-level observational study. eClinicalMedicine [Internet]. 2021 [cited 2022 Aug 4];42. Available from: https://www.thelancet.com/journals/eclinm/article/PIIS2589-5370(21)00479-X/fulltext
